# Incidental Left Ventricular Aneurysm Discovered after Chest Pain Following a Motor Vehicle Collision

**DOI:** 10.7759/cureus.5798

**Published:** 2019-09-29

**Authors:** Michelle Hernandez, Sanjiv Gray, Vibhav Kanyadan, Javier Rosario, Latha Ganti

**Affiliations:** 1 Emergency Medicine, University of Central Florida College of Medicine, Orlando, USA; 2 Surgery, University of Central Florida College of Medicine, Orlando, USA; 3 Miscellaneous, Wheeler High School, Marietta, USA; 4 Emergency Medicine, Envision Physician Services, Orlando, USA

**Keywords:** aneurysm, aneurysm, left ventricular aneurysm, chest pain, motor vehicle, vehicle collision, coronary artery disease, trauma, airbag

## Abstract

We report the case of a female with a history of coronary artery disease who came in with chest pain after a motor vehicle collision. Imaging revealed an incidental left ventricular aneurysm. The presentation and management of left ventricular aneurysms are discussed, along with imaging findings on computed tomography, plain radiography, and ultrasonography.

## Introduction

A left ventricular aneurysm is defined as a well-delineated, thin, scarred or fibrotic wall, devoid of muscle or containing necrotic muscle [1]. Left ventricular (LV) aneurysms are usually a result of a healed transmural myocardial infarction and occur in about three to five percentages of patients after myocardial infarction. Trauma, iatrogenic, Chagas disease, hypertrophic cardiomyopathy, and sarcoidosis are other reported causes [2]. Aneurysms of the apex and anterior wall are more than four times as common as those of the inferior or inferior-posterior walls. Clinical findings if present can overlap with those of other cardiac diseases. Dyskinetic movement of the left ventricle can lead to congestive heart failure and ST elevation in the electrocardiogram which is also seen in pseudoaneurysms [3]. Chest pain, dyspnea, and hypotension are common findings in left ventricular aneurysms. Sluggish flow can lead to the formation of thromboembolic events [2]. As a left ventricular aneurysm can have nonspecific symptoms, imaging is usually required for diagnosis and evaluation [4].

## Case presentation

A 64-year-old woman presented to the emergency department with chest pain after a car accident. She was the restrained driver, wearing a seatbelt, with impact on the driver's side and there was airbag deployment. She met trauma alert criteria and was brought in on a backboard and cervical collar. 

The patient did not have any loss of consciousness. She only complained of left lower back pain and chest pain. Her vital signs were: pulse 113 beats per minute, blood pressure 173/76 mm Hg, respiratory rate 16 breaths per minute, oxygen saturation 98% on room air, and temperature 97.9^0^F. On physical examination, the patient was alert and oriented with a Glasgow Coma Scale (GCS) of 15. Examination of the thorax revealed ecchymosis in a seatbelt distribution. Her medical history included coronary artery disease with a myocardial infarction seven years prior (unknown which artery), diabetes mellitus, hypertension, and hyperlipidemia.

**Figure 1:**
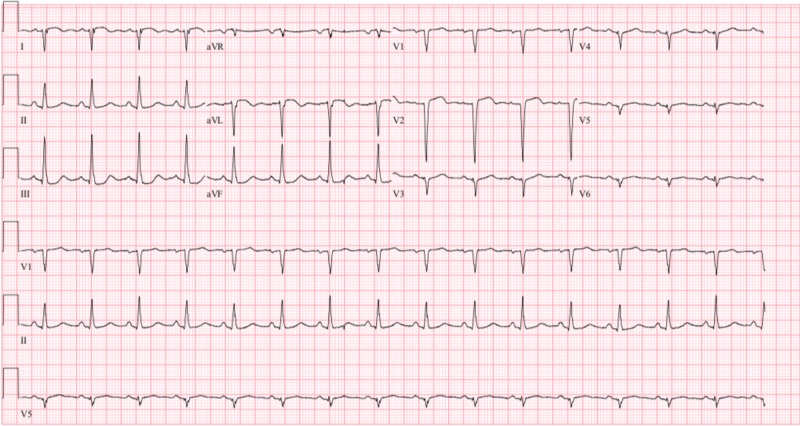
Patient's electrocardiogram demonstrating sinus rhythm, right axis deviation, and Q waves in the inferior leads without ST segment abnormalities.

**Figure 2:**
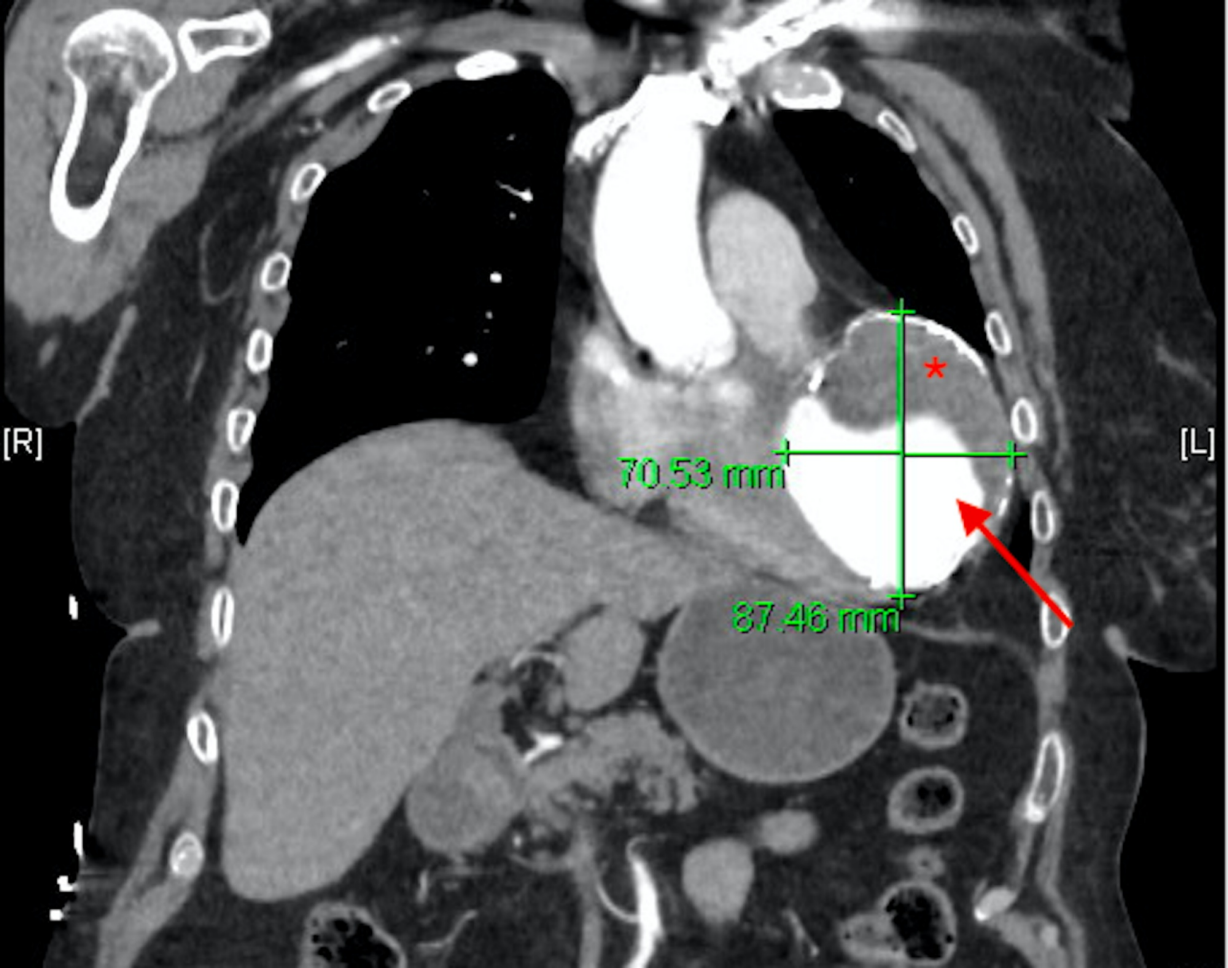
Contrast-enhanced CT, coronal section, shows a large opacity seen connected with the left ventricle extending down near the left hemidiaphragm (arrow). A mural thrombus is also seen within the aneurysm (asterisk).

**Figure 3:**
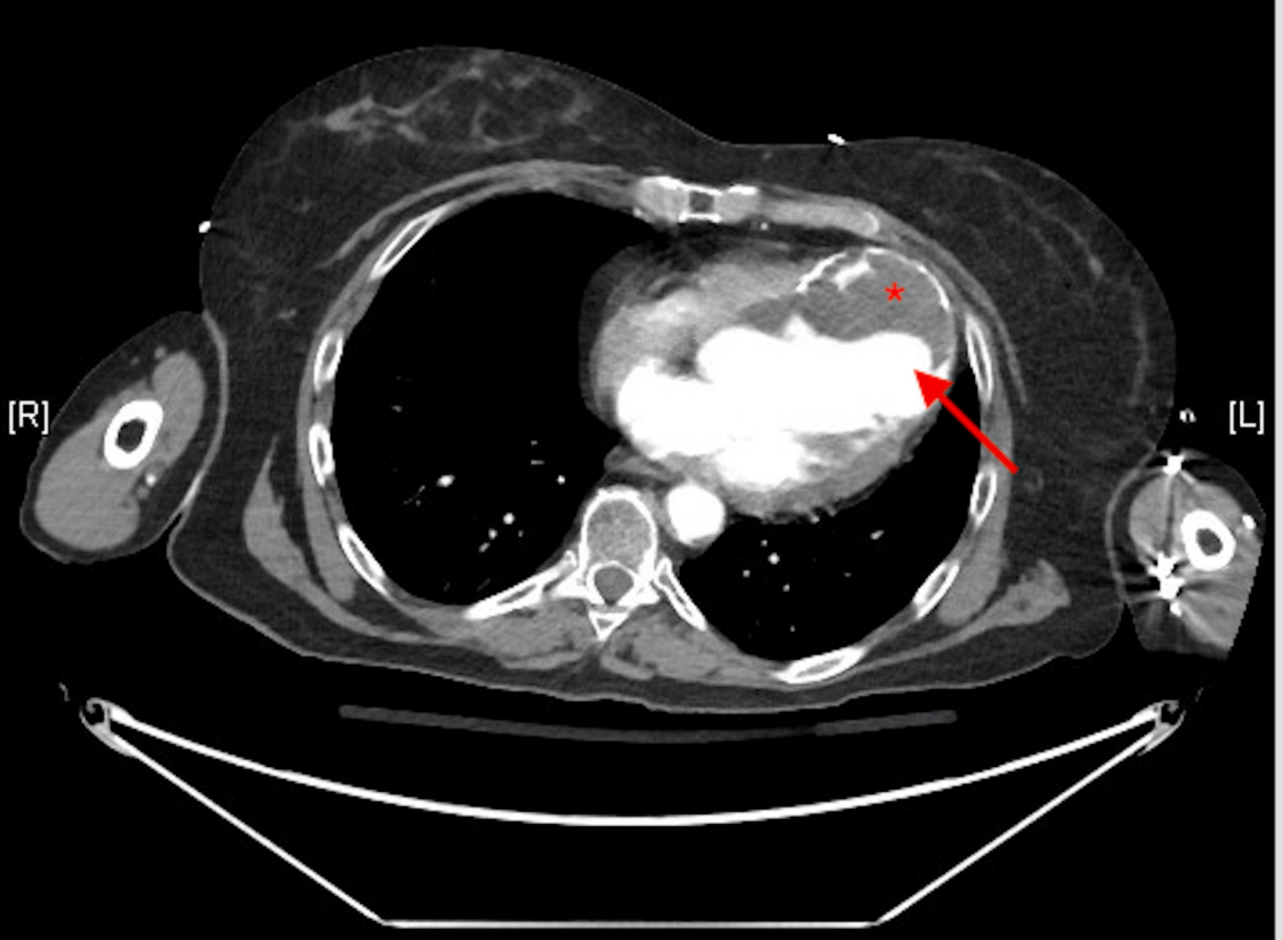
Contrast-enhanced CT, axial section - a large mass is seen adhering to the anterior left ventricular wall suggestive of a large mural thrombus (asterisk) within the left ventricular aneurysm (arrow).

**Figure 4:**
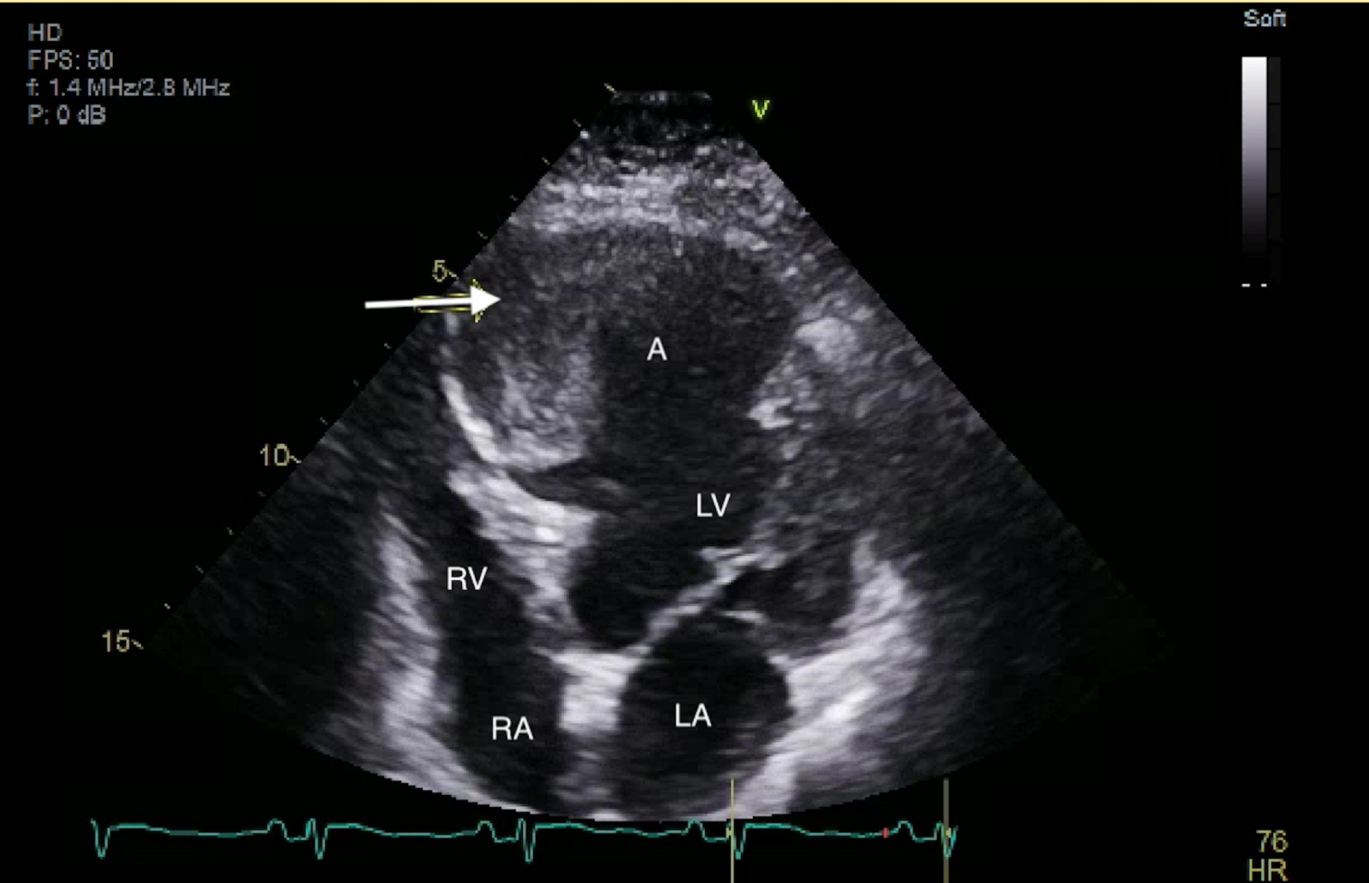
Ultrasonography confirmed aneurysmal dilatation of the left ventricle (arrow). RV - right ventricle; RA - right atrium; LV - left ventricle; LA - left atrium; A - aneurysm

**Figure 5:**
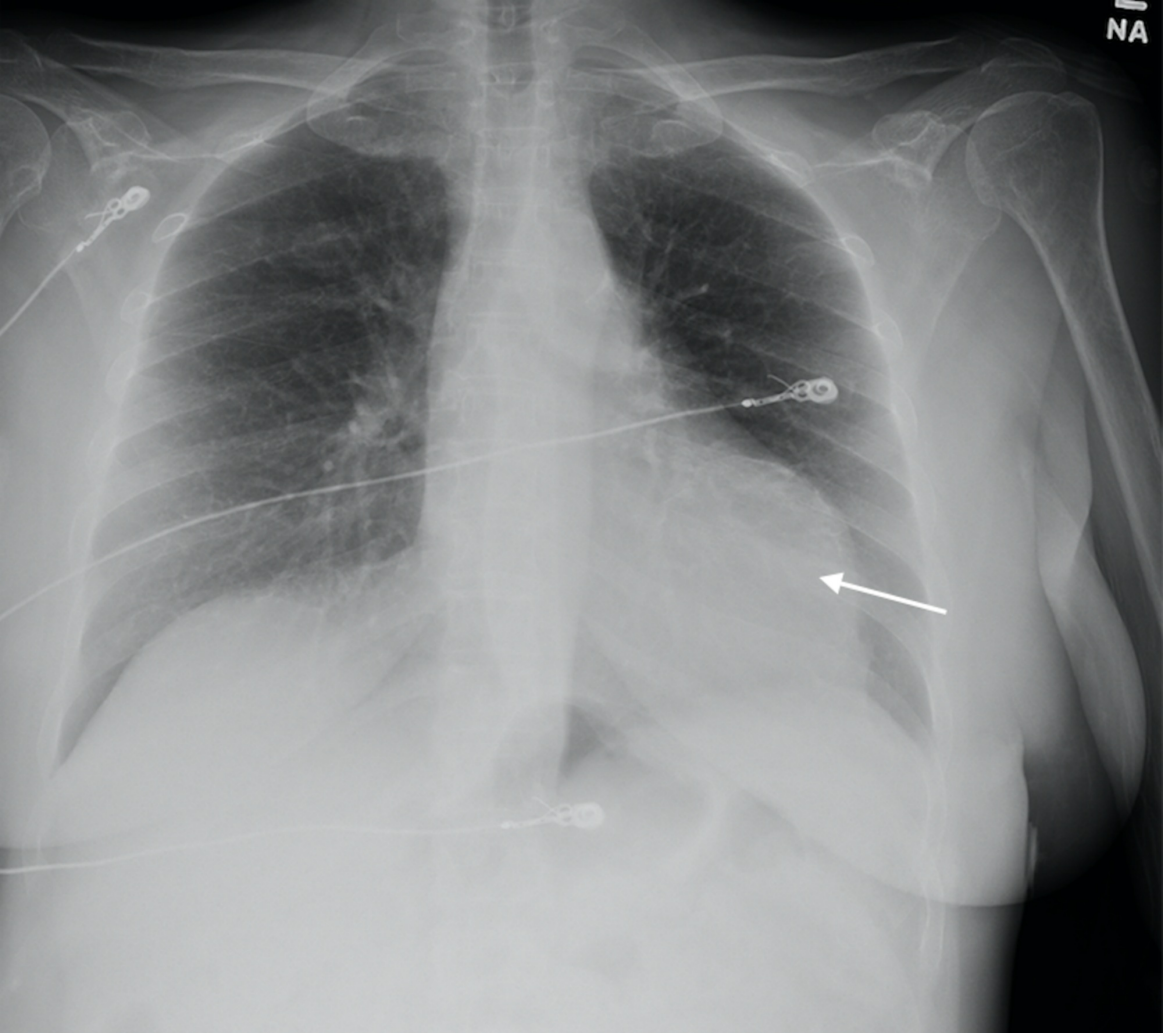
Chest radiograph demonstrated abnormal heart contour suggestive of aneurysmal dilatation (arrow).

Due to her complaint of chest pain, an electrocardiogram (ECG) and computed tomography angiography (CTA) of the chest and abdomen were done. The ECG (Figure 1) showed normal sinus rhythm, a right axis deviation, Q waves in the inferior leads without ST segment abnormalities. The chest CTA showed an incidental finding of a left ventricular aneurysm with a large amount of mural thrombus located within the aneurysm. Coronal CTA of the abdomen showed a large opacity seen connected with the left ventricle extending down near the left hemidiaphragm (Figure 2). On the axial view, a large mass is seen adhering to the anterior left ventricular wall suggestive of a large mural thrombus within the left ventricular aneurysm (Figure 3). Echocardiography confirmed aneurysmal dilatation of the left ventricle (Figure 4) with a reduced ejection fraction (15-20%) with regional wall abnormalities and chest radiograph (CXR) revealing an abnormal heart contour suggestive of aneurysmal dilatation (Figure 5).

Cardiothoracic surgery was consulted during hospitalization. No surgical interventions were considered necessary at the time as it appeared chronic, so she was instructed to follow up as an outpatient and to continue her current medications which include a Beta blocker, angiotensin-converting enzyme (ACE) inhibitor, and aspirin. 

## Discussion

Diagnostic dilemma

A true aneurysm results after thinning of the ventricular wall forming an out-pouching of the ventricle and a dyskinetic myocardium; a pseudoaneurysm occurs after free wall rupture of the left ventricle and it is contained by the surrounding pericardium. It does not contain all three normal arterial layers [3]. Diagnostic accuracy is crucial as a pseudoaneurysm usually requires emergent surgery. Transthoracic echocardiography (TTE) can be used to differentiate between the two. In our case, the myocardium is seen surrounding the thrombus, suggesting the presence of a left ventricle aneurysm. Limitations of the transthoracic echocardiogram include a limited field of view, operator dependence, and poor acoustic windows. Other imaging such as transesophageal echocardiography, a cardiac CT with ECG gating, cardiac magnetic resonance imaging (MRI), and coronary angiography may provide a better yield if the TTE is inconclusive. Cardiac MRIs with high spatial resolution and tissue characterization are ideal to distinguish an LV aneurysm from a pseudoaneurysm. However, not all patients can undergo MRIs because of either patient contraindications or center availability [3]. 

Treatment options

If ruptures are not noted and the myocardium wall integrity is confirmed it is likely a true left ventricular aneurysm, which can be treated medically and/or by elective surgery. Medical therapy includes an ace inhibitor for afterload reduction and anticoagulation if a thrombus is present. Surgical therapy may be indicated if refractory to medical therapy and is aimed at improving cardiac remodeling, diminishing heart failure, and improving survival. This is in contrast to a pseudoaneurysm which is important to detect early as emergent surgery is usually indicated [2]. Prompt and effective treatment of myocardial infarction can reduce the incidence of LV aneurysm all together [5].

In our patient, an incidental left ventricular aneurysm was seen; medical management was continued and close follow up with a cardiothoracic surgeon was advised. It is important to inform the patient of the possible major complications of left ventricular aneurysms, which include but are not limited to thromboembolism, ventricular arrhythmia, worsening congestive heart failure, refractory angina and possible cardiac rupture [1]. 

## Conclusions

Left ventricular aneurysms are not uncommon and sometimes can be an incidental finding unrelated to the condition for which a patient presented. It is important to recognize the appearance of a left ventricular aneurysm and distinguish it from a pseudoaneurysm as the treatment plan if often different. In this case, an incidental left ventricular aneurysm was discovered in a trauma patient. The patient was instructed to continue medical management with an ACE inhibitor, beta blocker, aspirin and close follow up with a cardiothoracic surgeon advised.
